# A Novel Variant in the TBC1D24 Lipid-Binding Pocket Causes Autosomal Dominant Hearing Loss: Evidence for a Genotype-Phenotype Correlation

**DOI:** 10.3389/fncel.2020.585669

**Published:** 2020-11-12

**Authors:** Thomas Parzefall, Alexandra Frohne, Martin Koenighofer, Juergen Neesen, Franco Laccone, Julia Eckl-Dorna, Jonathan J. Waters, Markus Schreiner, Sami Samir Amr, Emma Ashton, Christian Schoefer, Wolfgang Gstœttner, Klemens Frei, Trevor Lucas

**Affiliations:** ^1^Department of Otorhinolaryngology, Head and Neck Surgery, Medical University of Vienna, Vienna, Austria; ^2^Department for Cell and Developmental Biology, Orphan Disease Genetics Group, Center for Anatomy and Cell Biology, Medical University of Vienna, Vienna, Austria; ^3^Institute of Medical Genetics, Center for Pathobiochemistry and Genetics, Medical University of Vienna, Vienna, Austria; ^4^Rare and Inherited Disease Laboratory, London North Genomic Laboratory Hub, Great Ormond Street Hospital for Children NHS Foundation Trust, London, United Kingdom; ^5^Department of Biomedical Imaging and Image-Guided Therapy, Medical University of Vienna, Vienna, Austria; ^6^Laboratory for Molecular Medicine, Partners Healthcare Personalized Medicine, Cambridge, MA, United States; ^7^Department of Pathology, Brigham and Women’s Hospital and Harvard Medical School, Boston, MA, United States

**Keywords:** TBC1D24, DFNA65, genotype-phenotype association, exome sequencing, autosomal dominant, nonsyndromic, hearing loss

## Abstract

**Background**: Hereditary hearing loss is a disorder with high genetic and allelic heterogeneity. Diagnostic screening of candidate genes commonly yields novel variants of unknown clinical significance. *TBC1D24* is a pleiotropic gene associated with recessive DOORS syndrome, epileptic encephalopathy, myoclonic epilepsy, and both recessive and dominant hearing impairment. Genotype-phenotype correlations have not been established to date but could facilitate diagnostic variant assessment and elucidation of pathomechanisms.

**Methods and Results**: Whole-exome and gene panel screening identified a novel (c.919A>C; p.Asn307His) causative variant in *TBC1D24* in two unrelated Caucasian families with Autosomal dominant (AD) nonsyndromic late-onset hearing loss. Protein modeling on the *Drosophila* TBC1D24 ortholog Skywalker crystal structure showed close interhelix proximity (6.8Å) between the highly conserved residue p.Asn307 in α18 and the position of the single known pathogenic dominant variation (p.Ser178Leu) in α11 that causes a form of deafness with similar clinical characteristics.

**Conclusion**: Genetic variants affecting two polar hydrophilic residues in neighboring helices of TBC1D24 cause AD nonsyndromic late-onset hearing loss. The spatial proximity of the affected residues suggests the first genotype-phenotype association in *TBC1D24*-related disorders. Three conserved residues in α18 contribute to the formation of a functionally relevant cationic phosphoinositide binding pocket that regulates synaptic vesicle trafficking which may be involved in the molecular mechanism of disease.

## Introduction

Autosomal dominant (AD) nonsyndromic hearing loss (HL) is a heterogeneous condition regarding the age of onset, frequencies affected, progression, and severity. In total, 49 genes have been identified as causative to date (hereditaryhearingloss.org) that have a wide range of pleiotropy and function. The high genetic and allelic heterogeneity of HL poses a challenge to the clinical assessment of novel variants routinely encountered during the diagnostic screening. Identification of novel genes and variants not only expands diagnosis and genetic counseling but increases understanding of human hearing and deafness and can lead to personalized medicine approaches. Whole-exome sequencing (WES) and targeted next-generation sequencing of HL genetic panels have greatly facilitated the analysis of genetic HL in recent years. *TBC1D24*, coding for TBC1 Domain Family Member 24, is a pleiotropic gene that has been associated with autosomal recessive (AR) HL (DFNB86), ADHL (DFNA65), and a range of epilepsy syndromes (Mucha et al., 2017). Attempts to establish TBC1D24 genotype-phenotype correlations with AR variants have been unsuccessful, complicating the evaluation of variant pathogenicity. Here, we report a novel (c.919A>C; p.Asn307His) mutation in *TBC1D24* identified during WES and diagnostic panel screening of two unrelated ADHL families from Austria and the UK implying an association between AD HL disease phenotype and genetic variation affecting structural elements involved in phospholipid binding (Fischer et al., 2016).

## Subjects and Methods

### Patient Recruitment and Clinical Testing

Study participants were recruited at the Department of Otorhinolaryngology at the Medical University of Vienna, Austria, as part of an ongoing screening project for genetic HL and for diagnostic testing at the Great Ormond Street Hospital for Children, London, UK. Selected probands underwent speech audiometry, brainstem evoked response audiometry (BERA), otoacoustic emission (OAE) testing, video head thrust tests, vestibular caloric assessments, and neurocranial magnetic resonance imaging (MRI). Degrees of HL were defined as mild (20–40), moderate (41–70), and severe (71–95) in threshold dB values. The study met the WMA Helsinki Declaration criteria and was approved by the local Ethics Committee of the Medical University of Vienna (ECS 198/2004, with annual extensions to date). Informed consent was obtained from all participants.

### DNA Sequence Analysis and Variant Assessment

**Figure 1:**
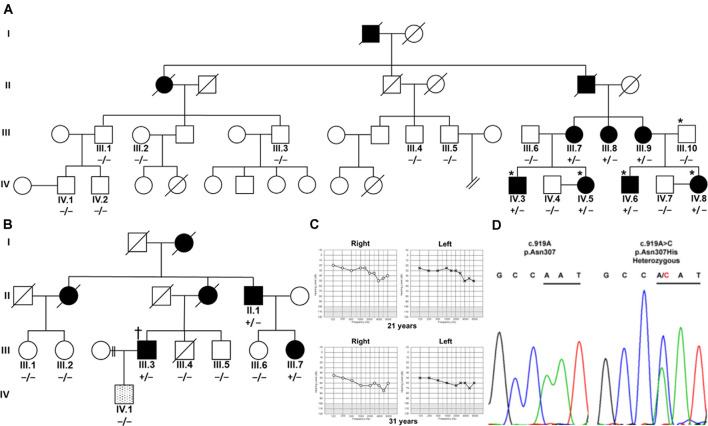
Segregation of TBC1D24 p.Asn307His with autosomal dominant (AD) nonsyndromic hearing loss (HL). **(A)** Four affected (shaded) family members and a normal-hearing (open) father in an Austrian family were analyzed by whole-exome sequencing (*). **(B)** In a UK family, one affected family member (†) was analyzed with panel sequencing. Segregation of heterozygous (+/−) c.919A>C in *TBC1D24* was tested in all available members of both families by Sanger sequencing. A single member of the UK family (lined; IV.1) developed a very-late-onset, predominantly mild high-frequency HL and did not segregate p.Asn307His. **(C)** Unaided, masked pure-tone audiograms in dB hearing levels of patient IV.8 at the ages of 21 and 31 years show progression to moderately severe pantonal HL. **(D)** Representative chromatograms show the A>C variation in IV.8 and the wildtype sequence in control subject IV.4. Codon 307 is underlined.

Chromosomal DNA was extracted from venous using with a commercial extraction kit (Invisorb blood universal kit 1000, STRATEC Molecular, Berlin, Germany) or the Chemagic STAR system (Perkin Elmer, Waltham, MA, USA) for British family members. Patients IV.3, IV.5, IV.6, and IV.8, and the normal-hearing father (III.10) in the Austrian family (Figure 1A) were selected for WES. Libraries were prepared with a commercial (SureSelectXT All Exon, V5) and a custom capture kit of 95 genes^1^ for the screening of the Austrian family and the UK proband (SureSelectXT Agilent Technologies, Santa Clara, CA, USA) respectively, followed by sequencing on an Illumina HiSeq 2000 (Austrian family) or MiSeq (UK proband) device (Illumina Biotechnology, San Diego, CA, USA). The UK sample was processed with an in-house bioinformatics pipeline, involving alignment with Burrows-Wheeler read aligner (BWA), variant calling with Freebayes, annotation with VEP95, re-annotation using Alamut batch, and variant analysis on an in-house system “GOSHG2P.” Reads from the Austrian family were mapped to the human genome reference build hg19 with the BWA (Li and Durbin, 2009) and variants were called with the Genome Analysis Tool Kit (Mckenna et al., 2010). All missense, deletion, and insertion variants in coding regions and splice sites were analyzed on an online massive parallel sequencing analysis platform (Genomatix GeneGrid, Genomatix GmbH, Munich, Germany) and visualized by interrogation of bam/bai files with the Integrative Genomics Viewer (IGV; Broad Institute, Cambridge, MA, USA). Variants were filtered at a maximum non-Finnish GnomAD European allele frequency of 0.00075 (Lek et al., 2016). A single common artifact in PPIAL4G (Gln24Leu; rs6604516) was removed by reference to a local WES database. All genomic positions listed refer to hg19. Candidate validation and segregation analysis were performed by PCR and standard Sanger sequencing. Primers were designed with Primer-BLAST interface^2^. All PCR reactions were performed in 2.5 mM MgCl_2_, 200 mM of dNTPs, 100 ng of chromosomal DNA, and 20 pmol of primers to amplify the segments of interest in *TBC1D24* (5′-CTTCCTGGCCTTTGAGTCGT-3′/5′-AGGATAGGACCCGATGTCCC-3′), *SYNE4* (5′-CGGGATGGGAAATGGGTTGA-3′/5′-CGAACACCTGGGTCAAAGGA-3′) and *CCNF* (5′-CCCTTCCTGCCTGTCATGTG-3′/5′-TGTGCTAGGAGACAGCAGTAGG-3′) in 35 cycles of denaturation (95°C, 30 s), annealing (60°C, 30 s), and elongation (72°C, 90 s). To exclude genes not expressed in the cochlea as HL candidates, expression data for adult mouse inner (IHC) and outer hair cells (OHC; Liu et al., 2014) cochlear HC, supporting cells (SC; Scheffer et al., 2015) and spiral ganglion neurons (Lu et al., 2011) at different developmental stages and overall inner ear soft tissue of 9-week-old mice (Geo GSE13421) were retrieved from the SHIELD^3^ and mouse cochlea gene^4^ databases. For FACS-sorted HC and SC data, the baseline for expression was set at a total of 15 reads from RNA-Seq across all samples (Scheffer et al., 2015) and at intensity levels of 10.9 for adult IHC and OHC microarray data (Liu et al., 2014). The isolated candidate variant was analyzed with SIFT^5^ and PolyPhen-2^6^.

### Protein Modeling and Cross-Species Alignment

The TBC domain was modeled on the Skywalker (human TBC1D24 equivalent residues 21–310; PDB 5HJN) and zebrafish TLDc domain-oxidation resistance protein 22 (residues 333–548; 4ACJ) crystal structures with the Protein Homology/analogY Recognition Engine V 2.0 (Phyre2^7^; Kelley et al., 2015). The PDB file of the generated model was analyzed in JMOL^8^ to illustrate bulkiness with Corey-Pauling-Koltun sphere representations and calculate atomic distances. TBC1D24 ortholog sequences from *Homo sapiens* (NP_001186036), *Pan troglodytes* (XP_016784129), *Rattus norvegicus* (NP_001099239), *Mus musculus* (NP_001157319), *Canis lupus* *familiaris* (XP_013970162), *Gallus gallus* (NP_001244200), *Xenopus laevis* (NP_001090574), *Danio rerio* (XP_009295920), and *Drosophila melanogaster* Skywalker (NP_610073.1) were aligned at Clustal Omega^9^ and visualized in AliView (University of Uppsala) with SeaView residue annotation.

## Results

### Clinical Phenotype in the Patients

Austrian (Figure 1A) and British Caucasian families (Figure 1B) were identified suffering from recurrent cases of nonsyndromic progressive ADHL. Onset was observed at an average age of 19 years 4 months ± 15 months for three members (IV.5, IV.6, and IV.8) of the Austrian family and 27 years ± 7.5 years in the British family (II.1, III.3, and III.7). HL developed from an initial mainly mild (below 2,000 Hz) to moderate (above 5,000 Hz) accentuated high-frequency HL slowly progressing to moderate-to-severe pantonal HL after 10 years (Figure 1C). Bone conduction results were not significantly different in the probands analyzed and no air-bone gap was observed (data not shown) consistent with sensorineural HL and excluding a conductive or mixed type of hearing loss. All affected family members use hearing aids for daily communication. Caloric reflex testing and video head thrust testing showed bilateral vestibular normoreflexia in all Austrian study patients. There was no history of syndromic neurologic disease, and brain MRI and neurological examinations showed no abnormalities in a member of the Austrian family (IV.8). One member (IV.1) of the UK family had grommets fitted as a child because of perceived hearing difficulties which were attributed to “glue ear.” At 35 years of age, he had normal hearing up to 4,000 Hz but began to develop a bilateral, predominantly mild, high-frequency HL. Due to the different clinical presentation compared to the other family members, a different cause of HL was considered likely.

### Genetic Screening Results

Patients IV.3, IV.5, IV.6, and IV.8 and the normal hearing father (III.10) in the Austrian family were selected for WES. Without utilizing coverage or quality filters, potential heterozygous pathogenic variants with a cutoff non-Finnish gnomAD European allele frequency (Lek et al., 2016) for a single heterozygous alteration at 0.00075 were identified in the genes *TPSG1* (16:1,272,028; NM_012467; c.726G>C; p.Trp242Cys; rs551902061), *TBC1D24* (16:2,547,068; NM_001199107.2; c.919A>C; p.Asn307His; submitted as rs1555501320) and *CCNF* (16:2,481,223; NM_001761.3; c.109G>A; p.Glu37Lys) at 16p13.3 and *SYNE4* (19:36,497,711; NM_001039876.3; c.559C>T; p.Arg187Ter; rs750797779). *TPSG1*, *TBC1D24*, *CCNF*, and *SYNE4* variants are all segregated by Sanger sequencing in the family as potential causes of HL (Figure 1D). *TPSG1* cochlear expression was below the threshold levels set for cochlear expression and was therefore not further considered as an HL candidate. In the UK family, a *TBC1D24* c.919A>C variant was identified as a sole candidate in targeted HL screening with a panel of 95 genes. The variant segregated in three affected members of the family by Sanger sequencing (Figure 1B). The c.109G>A *CCNF* and c.559C>T *SYNE4* variants identified in the Austrian family were not present in the affected UK family members shown by Sanger sequencing. The hearing-impaired family member IV.1 with a different clinical presentation carried the c.919A *TBC1D24* wildtype allele (Figure 1B). In contrast to p.Ser178Leu (non-Finnish European allele frequency 2/112,984; 0.000018), p.Asn307His is not listed in the gnomAD database. A single variant at p.307 (p.Asn307Ser; 1/111,448; 0.000009) is listed. The p.Asn307His variant was predicted to be probably damaging with Polyphen-2 (score 1.000, sensitivity 0.00, specificity 1.00) and tolerated (score 0.21) with SIFT. Concerning the American College of Medical Genetics and Genomics/Association for Molecular Pathology guidelines applied to ADHL (Oza et al., 2018), p.Asn307His would be classified as likely pathogenic based on the criteria PM2 (absent or ≤0.00002 in population databases), PS4_supporting (two unrelated probands with the variant) and PP1_strong (segregation in five affected relatives).

### Protein Model and Cross-Species Alignment

**Figure 2:**
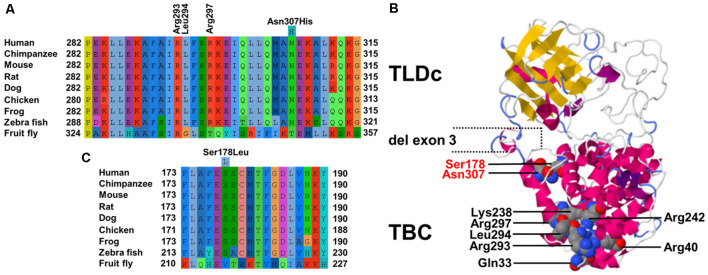
**(A)** Cross-species multiple alignments of the α18 helix of TBC1D24 (p.282–315) reveal high conservation of a polar uncharged residue at p.Asn307. The positions of the coordinating residues (p.Arg293, p.Leu294, and p.Arg297) responsible for binding phosphoinositides phosphorylated at positions 4 and 5 derived from the crystal structure of *Drosophila Skywalker* are shown **(B)**. Phyre2 model of human TBC1D24 based on crystal structures of Skywalker (human TBC1D24 residues 21–310; PDB 5HJN) and the zebrafish TLDc domain-oxidation resistance protein 22 (residues 333–548; 4ACJ). Corey–Pauling–Koltun sphere representations of mutated p.Arg307 and p.Ser178 amino acid positions in ADHL and all residues that contribute to the binding pocket are shown. Coordinates of the in-frame deletion of exon 3 in the shorter isoform are indicated. **(C)** Cross-species multiple alignments of the α11 helix of TBC1D24 (p.173–190) containing p.Ser178.

The TBC domain of TBC1D24 based on the homology model is predicted to contain the alpha-helical elements α1–18 (Table 1). Residue p. Asn307 in α18 is conserved in vertebrates (Figure 2A). Seven residues in helical elements α2 (p.Gln33 and p.Arg40), α15 (p.Lys238 and p.Arg242), and α18 (p.Arg293, p.Leu294, and p.Arg297) are human equivalent positions involved in the formation of a lipid-binding pocket recently identified in the *Drosophila* orthologue Skywalker. Cross-species alignments reveal full conservation of the residues p.Arg40, p.Lys238, p.Arg242 (data not shown), and p.Arg293 between human TBC1D24 and Skywalker. A single TBC1D24 variation (p.Ser178Leu) exchanging a highly conserved polar uncharged residue for a hydrophobic residue has been described previously in a European and a Chinese family by independent groups that cause AD HL with a similar age of onset and clinical course as the families under study (Azaiez et al., 2014; Zhang et al., 2014). The model predicts an interaction between p.Ser178 (α11) and p.Asn307 (α18) at an interhelix proximity of 6.8Å (Figure 2B). Cross-species alignments for α11 are shown in Figure 2C.

**Table 1:** Homology model predictions of TBC domain alpha helical elements in TBC1D24.

TBC alpha helix	Amino acid coordinates (p.)
1	12–21
2	32–40
3	48–59
4	62–67
5	73–81
6	90–95
7	103–105
8	108–124
9	134–144
10	148–160
11	173–190
12	192–200
13	206–219
14	222–234
15	238–253
16	254–259
17	265–274
18	282–315

## Discussion

Genetic testing revealed a novel rare missense (c.919A>C; p.Asn307His) variant in *TBC1D24* as the cause of disease in two unrelated families from Austria and the UK with late-onset, progressive, nonsyndromic ADHL. Whereas this variant was the sole candidate identified in the UK proband during diagnostic panel screening that segregated in all tested affected family members by Sanger sequencing, two further variations in *SYNE4* (c.559C>T; p.Arg187Ter) and *CCNF* (c.109G>A; p.Glu37Lys) co-segregated with the disease in the Austrian family screened by WES and Sanger sequencing. Although *SYNE4* variations have to date only been associated with ARHL, heterozygous p.Arg187Ter has been previously identified in a sporadic case of HL (ClinVar RCV000213729) with a heterozygous pathogenic ADHL *COCH* variation (p.Cys542Tyr; Yuan et al., 2008). We, therefore, could not completely exclude p.Arg187Ter as a modifier or a secondary driver in the development of AD HL in a single family. Since one missense (p.Ser178Leu) variant in *TBC1D24* has previously been shown to cause ADHL with similar clinical characteristics seen in this study (Azaiez et al., 2014; Zhang et al., 2014), the p.Asn307His variation (ClinVar registration VCV000425556.1) shared by both families was considered the cause of disease.

*TBC1D24* is a pleiotropic gene and variations cause both ARHL (DFNB86; MIM 614617), ADHL (DFNA65; MIM 616044), and several epilepsy syndromes such as AR DOORS (deafness, onychodystrophy, osteodystrophy, mental retardation, and seizures) syndrome (MIM 220500), AR early infantile epileptic encephalopathy-16 (MIM 615338) and AR familial infantile myoclonic epilepsy (MIM 605021; Mucha et al., 2017). To date, pathomechanistic investigations on TBC1D24 variations have been hampered by a lack of genotype-phenotype correlation when analyzing homozygous and compound heterozygous AR variants (Bakhchane et al., 2015; Aprile et al., 2019). Missense variations in the TBC domain and a truncating frameshift variation in the TLDc domain are known to cause prelingual-onset ARHL (Rehman et al., 2014; Bakhchane et al., 2015; Danial-Farran et al., 2018). Heterozygous carriers of the truncating variant are without symptoms (Azaiez et al., 2014; Rehman et al., 2017).

TBC1D24 is expressed in a broad range of tissues, and expression levels are highest in the brain (proteinatlas.org). In the auditory pathway, TBC1D24 is expressed in spiral ganglion cells, stereocilia and the cell bodies of IHC and OHC (Azaiez et al., 2014; Rehman et al., 2014; Zhang et al., 2014) although differences between murine and human expression patterns are still to be clarified. Tbc1d24 expression was reported in the SHIELD database in FACS-sorted supporting cells und HC from E16 to P7 and in spiral ganglion neurons from E12 to P15 but not in adult (P25-P30) IHC and OHC. In the Mouse Cochlea Gene Database, tbc1d24 expression was detected in whole cochleae of 9-week old mice. The 559-residue TBC1D24 protein contains an *N*-terminal Tre2-Bub2-Cdc16 (TBC) domain present in Rab GTPase-activating proteins (Rab GAPs), a domain linkage region, and a C-terminal TLDc domain with no known function but often found in TBC and lysine motif (LysM) domain-containing proteins (Corbett et al., 2010). A shorter 553-residue isoform (Figure 2B) generated by in-frame deletion of exon 3 (residues 322–327) is expressed in non-neural tissues (Guven and Tolun, 2013). Even though the presence of a TBC domain may indicate a role in vesicle trafficking, it is unknown whether TBC1D24 has Rab GAP activity as it lacks highly conserved residues pivotal for GTP hydrolysis (Pan et al., 2006; Hutagalung and Novick, 2011; Fischer et al., 2016). Recently, a lipid-binding pocket in TBC1D24 that binds phosphoinositides phosphorylated at positions 4 and 5 was identified from the crystal structure of the *Drosophila* ortholog Skywalker (human TBC1D24 equivalent residues 1–311) with the human TBC1D24 coordinates p.Gln33, p.Arg40, p.Lys238, p.Arg242, p.Arg293, p.Leu294 and p.Arg297 (Fischer et al., 2016). We, therefore, modeled human TBC1D24 on the Skywalker (residues 21–310; 5HJN) and the zebrafish TLDc domain-oxidation resistance protein 22 (residues 333–548; 4ACJ) crystal structures using Phyre^2^ (Kelley et al., 2015). Three binding-pocket equivalent residues (p.Arg293, p.Leu294, and p.Arg297) are located with p.Asn307 in α18 (Figure 2A). It is therefore conceivable that the p.Asn307His variant disturbs the integrity of the binding pocket. Interestingly, the wildtype model predicts an interaction between p.Ser178 (α11) and p.Asn307 (α18), the sole residues associated with ADHL to date, at interhelix proximity of 6.8 Å (Figure 2B). This proximity and interaction may be indicative of a novel genotype-phenotype correlation in DFNA65. An adjacent glutamic acid residue to p.Ser178 in α11 (Figure 2C) could also potentially interact with p.307 positively charged residue variants in α18.

In summary, we identify a second variation (p. Asn307His) in *TBC1D24* that causes ADHL. By generating a TBC protein model on the crystal structure of *Drosophila* Skywalker, we show that p. Asn307 is within predicted α-helix 18 that contains three residues involved in forming a lipid-binding pocket in Skywalker. In this model, p. Asn307 is in close interhelix proximity to p.Ser178 in α11, the single position identified to date to cause ADHL. Therefore, this is the first report of a probable genotype-phenotype correlation for *TBC1D24*-caused disease. The identification of potential interactions may not only enable the development of novel personalized therapies for HL but also has implications for genetic counseling. This is further illustrated by a report of pathogenicity for a negatively charged variation at codon 307 (p.Asn307Asp) that, in conjunction with an α18 missense variant c.845C>G (p.Pro282Arg), causes AR focal seizures, developmental delays, malignant migrating partial seizures of infancy, and hippocampal atrophy (Appavu et al., 2016).

## Data Availability Statement

The datasets presented in this study can be found in online repositories. The names of the repository/repositories and accession number(s) can be found in the article.

## Ethics Statement

The studies involving human participants were reviewed and approved by Ethics Committee of the Medical University of Vienna, Austria (ECS 198/2004). Written informed consent to participate in this study was provided by the participants.

## Author Contributions

EA, KF, TL, WG, JE-D, and TP: conceptualization. MK, JW, MS, AF, FL, JN, CS, TL, KF, and TP: methodology. JE-D, SA, JW, EA, AF, FL, JN, TL, and TP: data analysis. MS, SA, EA, FL, TL, CS, and WG: resources. SA, AF, KF, TL, and TP: data curation. AF, FL, TL, EA, and TP: preparation of the manuscript. WG, TL, and TP: supervision. JE-D, AF, MK, KF, CS, TL, and TP: project administration. WG, KF, CS, TL, and TP: funding acquisition. All authors have critically read and agreed to the published version of the manuscript. All authors contributed to the article and approved the submitted version.

## Conflict of Interest

The authors declare that the research was conducted in the absence of any commercial or financial relationships that could be construed as a potential conflict of interest.

## Footnotes

^1^www.labs.gosh.nhs.uk/media/529754/nsd_and_usher_v6.pdf

^2^www.ncbi.nlm.nih.gov/tools/primer-blast

^3^www.shield.hms.harvard.edu

^4^research.meei.harvard.edu/Otopathology/tbimages/mouse.html

^5^https://sift.bii.a-star.edu.sg/

^6^https://genetics.bwh.harvard.edu/pph2/

^7^www.sbg.bio.ic.ac.uk/phyre2

^8^www.jmol.org

^9^www.ebi.ac.uk/Tools/msa/clustalo
